# Supplementary material for: Bayesian refinement of protein structures and ensembles against SAXS data using molecular dynamics
Source: PLoS Comput Biol. 2017 Oct 18;13(10):e1005800. doi: 10.1371/journal.pcbi.1005800 (PMC5662244; doi:10.1371/journal.pcbi.1005800)
Supplement: S1 Archive — (BZ2) [file pcbi.1005800.s011.tar.bz2 › gromacs_4.62_bayesian_saxs_refi/share/html/online/g_xrama.html]

g\_xrama


| g\_xrama Main Table of Contents |

**VERSION 4.5  
Thu 26 Aug 2010**

---

### Description

xrama shows a Ramachandran movie, that is, it shows
the Phi/Psi angles as a function of time in an X-Window.

Static Phi/Psi plots for printing can be made with g\_rama.

Some of the more common X command line options can be used:  
-bg, -fg change colors, -font fontname, changes the font.

### Files

| option | filename | type | description |
| --- | --- | --- | --- |
| **-f** | traj.xtc | Input | Trajectory: xtc trr trj gro g96 pdb cpt |
| **-s** | topol.tpr | Input | Run input file: tpr tpb tpa |

### Other options

| option | type | default | description |
| --- | --- | --- | --- |
| **-[no]h** | gmx\_bool | no | Print help info and quit || **-[no]version** | gmx\_bool | no | Print version info and quit || **-nice** | int | 0 | Set the nicelevel || **-b** | time | 0 | First frame (ps) to read from trajectory || **-e** | time | 0 | Last frame (ps) to read from trajectory || **-dt** | time | 0 | Only use frame when t MOD dt = first time (ps) |

---

http://www.gromacs.org  
gromacs@gromacs.org
